# An Intensive Ambulatory Care Program for Adolescents With Eating Disorders Combining In-Person and Web-Based Care: Protocol for a Single-Site Naturalistic Trial

**DOI:** 10.2196/37420

**Published:** 2022-11-02

**Authors:** Kaylee Novack, Rachel Dufour, Louis Picard, Linda Booij, Nicholas Chadi

**Affiliations:** 1 Sainte-Justine University Hospital Research Center Montreal, QC Canada; 2 Department of Psychiatry and Addictology Université de Montréal Montreal, QC Canada; 3 Department of Psychology Concordia University Montreal, QC Canada; 4 Department of Psychology Sainte-Justine University Hospital Centre Montreal, QC Canada; 5 Department of Psychiatry McGill University Montreal, QC Canada; 6 Division of Adolescent Medicine Department of Pediatrics, Sainte-Justine University Hospital Centre Université de Montréal Montreal, QC Canada

**Keywords:** eating disorders, adolescents, ambulatory care, web-based care, telemedicine

## Abstract

**Background:**

The incidence of eating disorders (EDs) among adolescents has significantly increased since the beginning of the COVID-19 pandemic. Hybrid care, which combines web-based and in-person modalities, is a promising approach for adolescents with EDs but remains understudied in this population.

**Objective:**

We aimed to implement a novel hybrid (web-based and in-person) intensive ambulatory care program for youth and evaluate its feasibility, acceptability, and preliminary effectiveness.

**Methods:**

We will use a naturalistic pretest-posttest design to evaluate our proposed pilot Intensive Ambulatory Care Program (IACP). This novel type of day hospital care follows evidence-based principles and uses a family-centered, educational, and motivational approach. It will be tailored to the psychological needs of each participant and will be delivered in a hybrid format. A total of 100 participants meeting the DSM-5 (Diagnostic and Statistical Manual of Mental Disorders, Fifth Edition) criteria for EDs, aged 12-18 years, will be recruited over the 2-year trial period. We will examine recruitment, retention, and adhesion-to-protocol rates; participant and family satisfaction; and preliminary effectiveness using quantitative self-report questionnaires.

**Results:**

Rolling recruitment will take place from winter 2022 to fall 2023, during which time we expect to recruit approximately 80% (100/120) of eligible participants, retain at least 75% (75/100) of enrolled participants and have at least 70% (70/100) of enrolled participants complete at least one therapeutic session per week and all pre- and postintervention questionnaires. Data collection will occur concurrently. We base our recruitment and retention estimates on previous literature and consider that the highly flexible design of the IACP and the fact that no extra work will be required of individuals in the program to participate in the study, will lead to high levels of feasibility. We anticipate that participants and their families will be satisfied with both the program and hybrid delivery format. We expect that participation in the IACP will be associated with a medium effect size reduction in ED psychopathology from baseline to end of treatment. The data analysis and manuscript writing are expected to be completed by the summer of 2024.

**Conclusions:**

Given the high clinical burden associated with EDs, this study has the potential to fill an important research gap by testing the implementation of a novel hybrid mode of intervention. If feasible, acceptable, and effective, the IACP could lead to important improvements in health care services for adolescents with EDs.

**International Registered Report Identifier (IRRID):**

PRR1-10.2196/37420

## Introduction

### Burden of Eating Disorders

Eating disorders (EDs) are a group of serious and complex mental illnesses characterized by disturbed beliefs about body weight, shape, and image, in addition to maladaptive eating behaviors, including restriction, purging, and other methods of excessive compensation for caloric intake [1]. Although EDs can affect individuals of all ages, they often occur during adolescence [2-4]. Illness severity varies widely, and symptoms are highly heterogeneous. The negative consequences of these diseases include poor quality of life, impaired psychosocial functioning [5], psychiatric comorbidities [6], multisystemic medical complications, and mortality [7]. The treatment of EDs also varies widely. Hospitalization is generally reserved for patients at high medical or psychological risk who are unresponsive to other treatments, while ambulatory care is used for patients with less severe forms of illness [1].

### Day Treatment Programs for EDs

Day treatment programs provide patients with care on multiple days or hours per week, at an intensity that falls between hospitalization and ambulatory care [8]. This is important for patients with moderate to severe illness who either do not require hospitalization or who can benefit from step-down care after an inpatient hospitalization. The literature supports the use of day treatment programs, considering the limited benefits of extended hospitalization when compared with short hospitalization followed by prompt transition to ambulatory care [9]. The latter option may also reduce health care costs [10] and allow for a more rapid return to school and social functioning without affecting clinical outcomes [9]. Therapeutic approaches used in day programs vary but can be categorized as either family-focused [11-13] or nonfamily-focused [14-16] with most of the latter combining several modalities, including cognitive behavioral therapy, dialectical behavioral therapy, behavioral therapy, cognitive remediation therapy, and acceptance and commitment therapy, among others [8]. Short-term hospitalization followed by day program treatment for adolescents with AN is noninferior in terms of weight outcomes, 1-year rates of readmission, and ED symptoms when compared with continued inpatient treatment [17]. Similarly, several uncontrolled trials [18,19] and a systematic scoping review of the literature [8] suggest that day programs alone are effective in promoting weight gain for those who are underweight, decreasing ED and comorbid psychopathology, and improving psychosocial functioning and quality of life among individuals with moderate to severe ED symptoms.

Considering the high rates of comorbid mental health symptoms among youth with EDs [20], there is a need for integrated treatment strategies targeting both ED symptoms and psychiatric comorbidities. Day treatment programs may offer a unique opportunity to combine multiple treatment modalities because of their intermediate level of intensity and increased scheduling flexibility compared with inpatient treatment. In many cases, day treatment may also allow for the continuation of school and work-related activities. One example of this integrated approach, which included the addition of a self-esteem and social skills therapy group to a multidisciplinary ED day treatment program, effectively improved outcomes, such as happiness, satisfaction, and self-concept related to weight, shape, and others [21]. Day treatment programs may also be particularly amenable to personalized treatment plans given their flexibility, and some interventions that have used data-driven approaches to elaborate personalized plans have been found to be preliminarily feasible and acceptable [22,23].

### Web-Based Day Treatment Programs for EDs

The provision of day treatment remotely using technology has the potential to increase access to treatment by addressing barriers such as precautions for infection control (in the context of current or future pandemics) and geographic distance from urban centers (where in-person day treatment programs are typically delivered). However, the evaluation of web-based day treatment programs for youth with EDs has been identified as a research gap [24]. Limited evidence from a naturalistic study conducted during the COVID-19 pandemic to evaluate the experiences of youth transitioning from an in-person to a web-based day treatment program, suggests that this approach is acceptable for youth [25]. A recent scoping review [24] also found that therapy delivered via videoconference, including family-based treatment, cognitive behavioral therapy, and relapse prevention using the Maudsley Model of Anorexia Nervosa Treatment for Adults, may be effective in the ambulatory setting, although evidence was drawn from uncontrolled case reports, pilot trials, and feasibility trials.

In light of the increased need for services for adolescents with EDs [26-30] and the limited amount of empirical evidence for integrated treatment strategies combining in-person and web-based care for the treatment of pediatric EDs and comorbid psychopathologies, we are implementing and evaluating a pilot Intensive Ambulatory Care Program (IACP), a novel type of day treatment program. Our program will be tailored to the psychological needs of each participant and delivered in a hybrid format, both web-based and in-person. We describe the proposed intervention and methodology of a naturalistic study that will be used to evaluate its feasibility, acceptability, and preliminary effectiveness.

### Objectives and Hypotheses

The primary objective of this study is to describe the feasibility and acceptability of flexible, modular, and hybrid IACP for adolescents with EDs. Secondary aims include describing the baseline characteristics of the adolescents who enroll in the IACP, describing the preliminary effectiveness of the IACP for adolescents with EDs in an uncontrolled naturalistic setting, and describing the moderating role of age, ED diagnosis (eg, anorexia nervosa vs other ED diagnoses), length of illness, and level of attendance on clinical response to the IACP.

We hypothesize that recruiting and retaining participants in the IACP would be feasible and acceptable. We expect that participants will mostly present restrictive ED symptomatology, which is representative of the patient population seen in the ED clinic where the study will be conducted; participants will present comorbid symptomatology and ED-related behaviors, such as anxiety and depressive symptoms [31,32], high levels of affective reactivity [33] and perfectionism scores [34], poor coping [35] and self-esteem [36] skills, and high levels of social media use [37], as reported in the literature. We expect that participation in the IACP will be associated with a reduction in ED psychopathology, from baseline to the end of treatment, and that patient age, ED diagnosis, length of illness, family connectedness, and the number of hours of therapy attended will act as moderators of the preliminary effectiveness of the intervention. Although there is no consensus on moderators of treatment outcomes in the adolescent population, these moderators were chosen based on limited evidence from several reviews [8,38,39] that have identified individual, clinical, and family-related factors that are related to treatment response.

## Methods

### Overview

Our team will conduct a naturalistic study of the IACP for youth with EDs that will gather 3 types of data. First, the feasibility of the IACP will be evaluated using recruitment, retention, and adhesion-to-protocol rates. Second, the acceptability of the IACP program and web-based delivery method among youth participants will be measured using youth and parent satisfaction questionnaires. Finally, the preliminary effectiveness of the IACP will be described using quantitative self-report questionnaires pertaining to ED symptomatology, and several moderators of this effect will be investigated. Body mass index (BMI) and quantitative self-report measures of comorbid psychopathology will be used as secondary outcome measures to describe the preliminary effectiveness of the intervention in an uncontrolled naturalistic setting.

### Population

Individuals eligible for the study will be between 12 and 18 years of age, have a diagnosed ED according to the DSM-5 (Diagnostic and Statistical Manual of Mental Disorders, Fifth Edition) criteria [40], and will already have received medical treatment in the hospital or ambulatory setting at our specialized ED clinic. This clinic is located in a tertiary pediatric hospital in a large city in the province of Quebec, which serves both a multicultural urban population representative of other large cities in North America in addition to the surrounding suburban and rural populations. Individuals who meet these criteria will be invited to participate in the IACP and the current research. To be eligible for the IACP, individuals must be available to participate in all aspects of the proposed intervention (including pre- and postintervention measures, individual, family, and group sessions, and an intake and feedback meeting with the IACP clinical team).

Individuals will be excluded from the IACP (and the current research study) if they require hospitalization for medical stabilization when evaluated for recruitment. In addition, the intervention will be discontinued and the individual referred to the appropriate service if they become medically unstable during the program (eg, heart rate <45 bpm, body temperature <35.5 °C) or express acute psychiatric distress (eg, active suicidal ideation requiring hospitalization).

### Recruitment, Enrollment, and Consent

Clinicians working in ambulatory and hospital settings at our specialized ED clinic will refer patients who are suitable for the IACP. These patients will be screened for eligibility by an IACP clinical coordinator. All patients who meet the criteria for participation in the IACP will receive a brief explanation of the study during the initial screening meeting with the clinical coordinator and will be presented with the opportunity to meet a research assistant to discuss consent if they are interested in participating in the research study. It will be made clear that participation in the research study is optional and that it will not affect any care or services received in the IACP.

The meeting with the research assistant will take place with the eligible participant and at least one parent, either in-person or via the secure Teams videoconferencing platform. The research assistant will verbally explain the objectives of the study, the main procedures involved, and the potential benefits and risks of participation. The participant and their parents will be given time to ask questions and consider participating. If they agree, a consent form (summarizing the verbal explanation of the study) will be presented to them for both parents and participants to sign.

Recruitment will occur on an ongoing basis over the 2-year study period. We expect that approximately 100 patients will be recruited (see the section on power calculation below for more details).

### Attrition and Compliance

We expect to recruit approximately 80% (100/120) of eligible participants—that is, 80% of all youth enrolled in the IACP—to our study, which is a conservative estimate based on previous literature [41]. Participation in the research study entails no additional commitment from eligible participants, given that all study materials are also an integral part of the IACP. Further, we expect attrition to be less than or equal to 25% (25/100), based on the current literature, which suggests a 7% to 42% dropout rate for adolescents enrolled in day treatment programs for the treatment of EDs [17,42-45], and also considering the flexible, modular, and hybrid nature of our program, which may allow us to improve retention rates.

Participants who attend at least one session per week of treatment in the IACP (and complete both pre- and postintervention materials) will be considered sufficiently exposed to the intervention and, thus, will be included in the data analysis on the preliminary effectiveness of the program.

### Study Design

**Figure 1:**
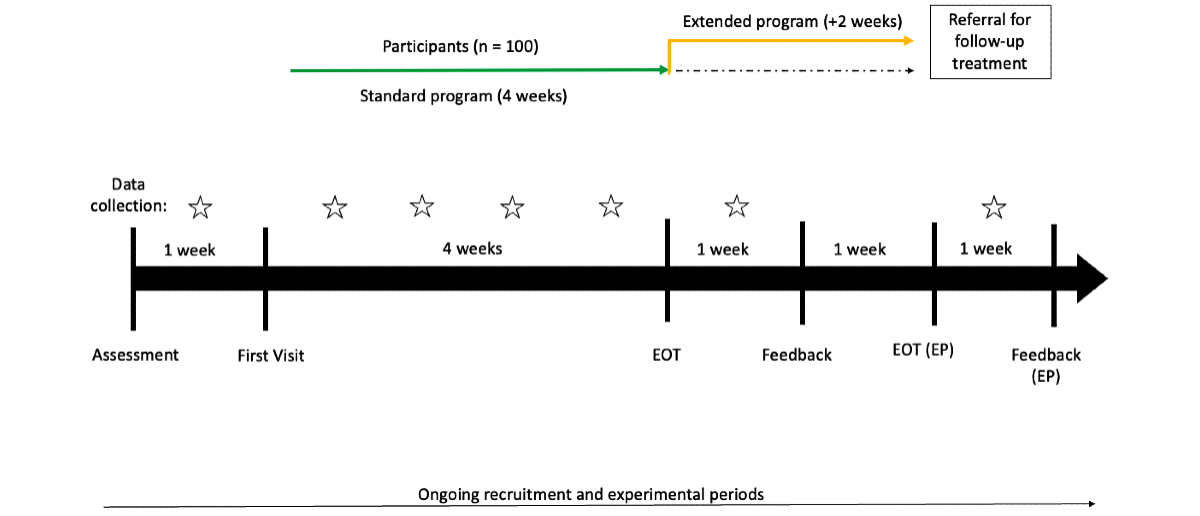
Experimental design of the 6- to 8-week IACP for adolescents (12-18 years) with eating disorders. EOT: end of treatment; EP: extended program.

This study will have a natural design. Therefore, all participants who consent to the trial will receive the same individualized treatment modality as part of the IACP. The study will be an uncontrolled, pre- and posttest trial (Figure 1), comparing measures collected at baseline to measures collected at the end of treatment within participants.

### Data Collection

Data will be collected at baseline (clinical and demographic information and preintervention self-report questionnaires), weekly (youth and parent satisfaction questionnaires), and immediately after the end of treatment (postintervention self-report and satisfaction questionnaires), as outlined in Figure 1. The clinical program coordinator will be responsible for collecting all data, deidentifying the data, and sending all deidentified data (including only participants’ random study ID) to the research team. Data to be collected include demographic and clinical information at baseline, growth curve charts, BMI pre- and postintervention, self-report standardized outcome questionnaires, acceptability (satisfaction) surveys, and measures of feasibility. All self-report questionnaires and surveys will be completed either on paper (if participants are present on site) or web-based (on a laptop or smartphone) using password-protected interactive PDF format questionnaires that will be sent to the participants using a secure email address.

Demographic and clinical information at baseline, including age, sex, level of education, diagnosis, presenting ED symptoms, duration of illness, ED treatment history (including past hospitalizations), maximum and minimum weight, comorbid symptomatology, and past medical history, will be collected by the clinical program coordinator during the intake visit for the IACP.

BMI will be calculated at baseline and at the end of treatment. Weight (with clothing on but without coats, shoes, boots, or cold weather accessories) and height (without shoes) will be measured during the first and last appointments with the clinical team using an electronic scale and a standard wall measuring scale. BMI will be calculated by dividing body weight in kilograms by the square of the height in meters.

Feasibility data will be collected by the IACP clinical staff throughout the duration of the study and will include recruitment and retention rates, in addition to measures of adherence to the protocol (Table 1).

**Table 1:** Summary of feasibility measures.

Outcome	Target
Recruitment rate	80% (100/120) of eligible participants (ie, of all adolescents participating in the IACP^a^) will enroll in the study and complete the baseline measure.
Retention rate	≤25% (25/100) of enrolled participants lost to follow-up.
Adherence to protocol	At least 70% (70/100) of participants complete at least one therapeutic session per week of treatment in the IACP. At least 70% (70/100) of participants complete all pre- and postintervention questionnaires. At least 70% (70/100) of participants complete at least one therapeutic session per week and all pre- and postintervention questionnaires.

^a^IACP: Intensive Ambulatory Care Program.

### Intervention

The intervention uses a family-centered, educational, and motivational approach that is based on the biopsychosocial model of EDs as described in Aimé and Bégin [46]. This model theorizes that a combination of sociocultural, environmental, familial, and individual factors, in addition to biological predispositions, converge to contribute to both the development and maintenance of disturbed beliefs and behaviors that characterize EDs. The therapeutic and psychoeducational modules (Table 2) were created based on this theory [46] and on a variety of similar sources such as books by Daniel J Siegel on mindfulness and by Martha M Linehan on impulse control [47], on cognitive behavioral and psychoeducational activities that are carried out in numerous ED treatments (eg, an open letter to my anorexia, metaphors for change, etc), and on original material created in partnership with patients.

Each participant will have an individualized treatment plan combining one or more modules, which will be established based on the initial questionnaire evaluation results and discussions between the clinician, the participant, and their family. Modules on one or more of the following themes will be presented as follows: (1) EDs and related psychopathology; (2) mental health and emotional regulation; (3) stress and anxiety management; and (4) identity, relationships, and life cycle issues in adolescence. Parents will be invited to participate in interventions pertaining to the physical and psychological components of EDs, meal accompaniment, stress, anxiety, hyperactivity management, and family life.

The intake and evaluation process, which will guide the creation of an individual treatment plan, will take place over 1 week. Following this, the intervention will take place over 4 weeks, with the possibility of extending it by 2 weeks, based on a discussion with the clinical team and the participant’s individual needs and progress. The intervention will conclude with 1 week of feedback and evaluation. Overall, the programming will last for 6 to 8 weeks.

**Table 2:** Overview of the 4 therapeutic modules and corresponding activities and interventions offered within the Intensive Ambulatory Care Program.

Module	Activities and interventions
EDs^a^ and related psychopathology	Virtual meal accompaniment Hyperactivity management ED recovery and sources of motivation Perfectionism Body image
Mental health and emotion regulation	Recognizing emotions Emotion regulation Impulsivity and anger Suicidal and parasuicidal behaviors
Stress and anxiety management	Recognizing emotions Mindfulness Relaxation techniques Psychoeducation about stress
Identity, relationships, and lifecycle issues in adolescence	Changes during adolescence and fear of growing up Self-esteem and self-affirmation The influence of social media Relationships with parents and friends Communication

^a^ED: eating disorder.

The time commitment for program participants will be variable but will involve a minimum of two to three 60- to 90-minute sessions per week, including meal accompaniment and preparatory activities with the adolescent and at least one parent or guardian. This represents a total of approximately 3 to 4 hours of programing per week. This will be in addition to regular planned outpatient clinical appointments with doctors, psychologists, social workers, etc. which will be considered usual care and which are not part of the IACP.

The modules of the intervention will be delivered using various formats, including in-person and web-based individual meetings, in-person and web-based meetings with parents, web-based synchronous therapy activities (eg, relaxation or mindfulness) administered by clinicians, individual web-based asynchronous therapy activities completed alone by participants with web-based feedback provided by clinicians, web-based viewing of prerecorded informational videos, and participant and family completion of personal logbooks and assignments. Each module has a set format (eg, individual vs group; in-person vs web-based) and was developed by the clinical team based on clinical experience and relevant literature.

### Outcome Measures

A set of standardized questionnaires (Table 3) will be administered before the start of the IACP. Many of these will be repeated after the completion of the program (Table 3). The total time required for the administration of all questionnaires will be approximately 55 to 65 minutes at baseline and 40 to 50 minutes at the end of the treatment. The total time dedicated to pre- and postintervention questionnaires may seem long but is justified by their key importance to the development of individualized treatment plans and the importance of postintervention feedback and debriefing sessions with clinical staff. All questionnaires will be administered in French, given that the language of treatment at the study site is French, and that French is the official and the most spoken language in the province where the study will be held. Language preferences will be discussed at the initial screening meeting with the clinical coordinator, and participants will have the option to request to complete the questionnaires in English at this time.

**Table 3:** Psychometric properties and characteristics of the included questionnaires.

Questionnaire			Themes covered		Number of questions		Available research on validity^a^		Available research on reliability		Time necessary to complete		Time at which survey is completed (T_0_^b^; T_1_^c^)
**Primary outcome (eating disorder symptoms)**													
	Eating Disorder Examination Questionnaire—Adolescent version	Eating disorder symptoms		36 items		Adolescent (11-18 years old) [48]		Good internal consistency [48]		7-8 min		T_0_, T_1_	
**Secondary outcomes (comorbidities and associated behaviors)**													
	Affective Reactivity Index	Chronic irritability		6 items		Adolescent (3-18 years old) [49,50]		Good internal consistency [49]		1-2 min		T_0_, T_1_	
	Child and Adolescent Perfectionism Scale	Trait perfectionism		22 items		Adolescents (10-17 years old) [51]		Good internal consistency [51]		3-4 min		T_0_, T_1_	
	Patient Health Questionnaire for Adolescents	Depressive symptoms and suicidality		13 items		Adolescents (grade 8-12) [52]		Good internal consistency [52]		2-3 min		T_0_, T_1_	
	Revised Children’s Anxiety and Depression Scale	Anxiety and depression		47 items		Adolescents (English version: 8-13 years old [53]; French version: 10-19 years old [54])		Good internal consistency [53]		15 min		T_0_, T_1_	
	Generalized Anxiety Disorder 7	Generalized anxiety		7 items		Adolescents (English version: 14-18 years old [55]; French version 18-75 years old [56])		Excellent internal consistency [57]Good test-retest reliability [57]		1-2 min		T_0_, T_1_	
	Adolescent Coping Scale (Échelle de coping pour adolescents)	Coping strategies		79 items		Adolescents (English version: 12-18 years old [58]; French version: 14-17 years old [59])		Good internal consistency (French version) [59]		11-13 min		T_0_	
	Self-Esteem Rating Scale, short form	Self-esteem		20 items		Adults (English version: mean 26.8, SD 9.9 years [60]; French version: mean 24, SD 7.4 years [60])		Good internal consistency (French) [60]		3-4 min		T_0_, T_1_	
	Eating Disorder Recovery Self-Efficacy Questionnaire-French	Confidence regarding eating disorder recovery		23 items		Adults (English version: mean 26.3, SD 11.1 years [61]; French version: mean 21.8, SD 3.9 years [62])		Excellent internal consistency [61] Good test-retest reliability [62]		5-6 min		T_0_, T_1_	
	Dépistage/évaluation du Besoin d’aide—internet^d^	Problematic internet usage		15 items		Adolescents (16-29 years, mean 19.7 years [63])		No data available		3-4 min		T_0_	
	Hyperactivity questionnaire (*Questions pour évaluer le surexercice*)	Excessive exercise habits		3 items		No psychometric data available^e^		No psychometric data available^e^		1-2 min		T_0_, T_1_	
	Family Connectedness Questionnaire (*Fonctionnement familial*)	Family functioning		6 items		No psychometric data available^e^		No psychometric data available^e^		1-2 min		T_0_, T_1_	
Total response time			N/A^f^		N/A		N/A		N/A		55-65 min 40-50 min		T_0_ T_1_

^a^For the purposes of this study, we consider standardized questionnaires to be validated if they have shown favorable psychometric profiles in peer-reviewed studies.

^b^T_0_: baseline

^c^T_1_: end of treatment

^d^Three additional, nonvalidated questions were added to determine problematic use of the internet to access information about (1) food and calories, (2) exercise and energy expenditure, and (3) dieting and other ways of losing weight.

^e^No psychometric data were available for questionnaires created by members of our research team. However, these questionnaires were either based on relevant literature or used in other studies. Further details are provided in Multimedia Appendix 1 [25,34,49-92].

^f^N/A: not applicable.

The primary outcome measure for this study is the Eating Disorder Examination Questionnaire for Adolescents (EDE-A) [93], a 36-item self-report questionnaire that evaluates attitudes, feelings, and behaviors related to eating, body image, and weight. It is a close adaptation of the Eating Disorder Examination Questionnaire (EDE-Q) [64], the adult version of this questionnaire, which contains 28 items and is one of the most commonly used ED symptom scales [8,94,95]. The EDE-A was adapted to measure symptoms on a shorter timescale than the EDE-Q (14 days rather than 28 days), which was considered more developmentally appropriate by experts with experience in treating EDs in the adolescent population [93] and is better suited to our study given the short length of our intervention (4-6 weeks, excluding pre- and postintervention meetings). Like the EDE-Q, the EDE-A questionnaire yields a global score in addition to four subscale scores: restraint, eating concern, weight concern, and shape concern. Norms exist for EDE-Q scores in healthy [93] and clinical adolescent populations [48].

No studies have evaluated the internal consistency of the EDE-A specifically, however, the EDE-Q, on which it is based, has good internal consistency, with a Cronbach α of .96 in a sample of female adolescents with anorexia nervosa [48], and between .78 and .93 in the general population [96,97]. Our team produced an adapted version of the questionnaire using a validated translation of the EDE-Q by Turgeon [98] as a guide, given that no validated translations of the EDE-A were available.

The secondary outcomes are outlined in Table 3 and include measures of comorbid ED psychopathology and related behaviors, including anxiety and depression symptoms, irritability, perfectionism, self-esteem, coping skills, social media use, and family connectedness. Further descriptions of the secondary outcome measures, including their psychometric properties, can be found in Multimedia Appendix 1 [25,34,49-92].

Satisfaction questionnaires (acceptability) will consist of self-report surveys completed by the participants both weekly and at the end of the intervention. Weekly surveys will evaluate satisfaction with individual therapeutic activities (eg, meal accompaniment sessions, individual and group sessions) experienced in the IACP using Likert-type questions (eg, Was this activity interesting and useful? Did it help participants understand themselves or find solutions? Did participants feel understood? Were participants satisfied and engaged?) as well as open-ended questions about what participants liked, disliked, and thought were the most important takeaways from each intervention. Postintervention satisfaction surveys for parents and patients will evaluate overall program satisfaction and satisfaction with the web-based mode of intervention, using 10-point Likert-type and open-ended questions.

### Power Calculations

Sample size estimations were performed using G*Power 3.1. Sample size estimations were performed for all analyses, and the final targeted sample size was selected so that the analysis requiring the largest number of participants could be adequately powered.

As our proposed treatment is new and the goal of this project is to collect initial data on its effectiveness, formal power analysis cannot be conducted. However, based on the literature on the effectiveness of specialized ED care [13-15,99-104], we expect a medium effect size (f^2^=0.15). Recent work in adult patients in a specialized tertiary care ED program also showed that fully web-based care and fully in-person outpatient care both yielded a similar medium effect size [105]. Thus, we expect our hybrid model to yield medium effect sizes. This implies that the sample size required to reach a level of significance of P=.05 with a power of 0.80 is 68 participants. By testing three moderators and with an assumed dropout rate of 25% [17,42,43], we would need to recruit 98 people to test our hypotheses. Therefore, a total of 100 patients will be recruited.

### Statistical Analysis

Statistical analyses will be performed using SPSS version 27.0. Feasibility and acceptability will be analyzed by summarizing quantitative data from (1) clinician-reported recruitment rate, retention rate, and adherence to protocol using descriptive statistics; (2) weekly satisfaction surveys of individual and group activities; and (3) postintervention surveys of overall satisfaction and satisfaction with the web-based mode of intervention. Qualitative data from both the weekly and overall satisfaction surveys will be analyzed using conventional content analysis. Patient characteristics at baseline will be summarized using descriptive statistics.

The preliminary effectiveness of the IACP intervention will be examined using general linear mixed models, with changes in global EDE-A scores from baseline to the end of treatment as the primary outcome. Appropriate covariates (eg, number of attended sessions) and random factors (eg, therapist) may be added to the statistical model for exploratory analysis. Similar general linear mixed model analyses will be run for the secondary outcome measures (Table 3), comparing scores from the baseline to the end of treatment. Finally, general linear mixed models will also be used to study the moderating role of the level of attendance and other clinical and psychosocial factors such as age, length of illness, ED diagnosis (anorexia nervosa vs other types of ED), and family connectedness on the primary outcome (change in EDE-A scores from baseline to end of treatment).

### Ethics Approval and Participant Safety

The Scientific Committee of the Sainte-Justine University Hospital Center Ethics Committee (FWA00021692), which was designated by the Quebec government (Ministère de la Santé et des Services Sociaux du Quebec) in Montreal, reviewed and approved the study protocol (project ID number: 2022-3925). Concerning the intervention itself, the risks involved are minimal and inherent to participating in therapy, such as being confronted with difficult information regarding one’s own mental health, behaviors, attitudes, etc, which can lead to stress or anxiety. However, this is a part of the treatment process and is expected to lead to positive therapeutic outcomes. The risks related to the evaluation of the intervention are minimal. There will be no inconveniences in terms of travel time and time spent responding to questionnaires other than that required for the participants’ normal follow-up in the IACP. If participants disclose worrisome information in the questionnaires, especially those related to suicidal ideation, the clinical program coordinator (a clinical psychologist) will contact them promptly to provide appropriate support. The clinical program coordinator will refer the participants to the necessary services to ensure their safety. The participants will be notified in advance that the clinical program coordinator may disclose this information to their parents or caregivers. Risks related to data and information-sharing with the research team as well as the measures in place to maintain participant confidentiality (deidentification of all data, transfer of data via a secure email account, and data storage on secure hospital servers) will also be discussed with all participants and parents during the intake meeting with the clinical coordinator.

## Results

Recruitment for the study and data collection will be conducted on a rolling basis from winter 2022 to fall 2023. The data analysis and manuscript writing are expected to be completed by the summer of 2024.

## Discussion

### Anticipated Outcomes

We anticipate that our study will demonstrate the feasibility of running an innovative hybrid (web-based and in-person) IACP for adolescents in a specialized ED clinic located in a tertiary care hospital in a large urban center. We also anticipate that the intervention will be acceptable to both participants and their parents. We anticipate that the intervention will lead to a reduction in ED psychopathology, and that greater levels of participation in the IACP will be associated with a greater reduction in symptoms. We anticipate that participants recruited to participate in the study will represent a subset of the youth population with EDs on the more severe end of the disease spectrum (as patients with less severe illnesses would be less likely to be referred to the specialized ED program by their treating physician). Therefore, we anticipate that most study participants will present with severe ED symptoms (as measured by the EDE-A), comorbid symptoms of anxiety and depression, and personality traits predisposing them to perfectionism and low self-esteem, as reported in the literature [106,107].

### Future Implications

The IACP could represent a novel mode of treatment in terms of content, therapeutic approach, and mode of delivery, and would present important advantages for accessibility and patient-centered care, given its flexible and hybrid (in-person and web-based) nature. Indeed, the intent is to make the program as accessible as possible by removing barriers such as geographic distance and interference with school and family functioning.

If the results of this study show that such an approach is feasible, acceptable, and preliminarily effective, the model can be easily applied at other sites or in a larger population for a few reasons. First, the initial and final assessments used standardized questionnaires with favorable psychometric properties in child and adolescent populations. Second, guidelines for individualized treatment module selection will be created, allowing clinical coordinators to use predefined threshold scores from baseline assessments to elaborate treatment plans. Similarly, guidelines for evaluating whether participants should participate in regular or extended programs will be created.

### Strengths and Limitations

Our study protocol has several strengths. First, the outcome measures will be completely integrated into regular clinical evaluations of patients participating in the IACP. As such, participants and their families will not be required to spend any additional time participating in the study. Second, the novel hybrid model of treatment will facilitate the incorporation of sessions into families’ schedules and is flexible and adaptable to individuals’ living situations, favoring both participation in the IACP and study completion. Third, the treatment program will be individualized and tailored to each participant’s needs. This will ensure that participants receive treatment that focuses on the most pressing issues related to their ED. Given the alignment of treatment modules and standardized pre- and postintervention questionnaires, data analysis is likely to capture the most salient changes in symptomatology. Fourth, building on expanding literature, a comprehensive battery of questionnaires and outcomes will allow for meaningful analyses of several contributing factors related to the treatment of EDs in adolescents by making optimal use of several validated questionnaires. Finally, participants with a broad range of ED diagnoses will be included to appropriately represent diverse symptom presentations.

This study has a few limitations. First, being a single-site study, recruitment will be limited to the number of patients receiving care at the study site, which may limit the final sample size and generalizability of our findings. However, it should be noted that the study will be conducted in the largest tertiary pediatric care hospital in Quebec, a Canadian province with a population of 8.5 million inhabitants. Therefore, the results of this study can be generalized to other sites in large North American urban centers. Second, the naturalistic trial design and individualized treatment approach will make it so that some of the analyses and conclusions may be impacted by external confounders (such as changes in primary treatment, seasonality, and external stressors). However, it will allow for a better understanding of the real-world feasibility and acceptability of this type of day treatment program for EDs in adolescents. Third, given the highly flexible and personalized nature of the IACP, it will not be possible to compare the feasibility and preliminary effectiveness of in-person vs web-based treatment modules. However, acceptability data (satisfaction questionnaires) may provide important clues to the participants’ appreciation of the in-person and web-based components of the program. Finally, the short duration of follow-up in this project will not allow for long-term assessment of the effectiveness of treatment in reducing ED symptomatology. Future work may include long-term follow-up of youth participating in the IACP, as well as more detailed analyses on the effectiveness of different components of the program (eg, in-person vs web-based modules).

### Conclusions

Given the high incidence of EDs in the adolescent population and the important physical, psychological, and social impacts of these illnesses, research on scalable and adaptable treatment programs is crucial. Evaluating the feasibility, acceptability, and effectiveness of intensive ambulatory treatment delivered in a hybrid model is in line with this objective. Furthermore, the intervention we describe, using a hybrid and family-focused modular approach that adapts treatment to individual participants, has seldom been described in the existing literature. Despite its limitations, the findings of this study will help evaluate and refine our hybrid (in-person and web-based) IACP in real-life practice. It will also allow us to gain a better understanding of which patients could benefit from it the most. It is our hope that our study may help inform and improve the care of patients with EDs, both in our center and in other centers worldwide.

## Appendix

Multimedia Appendix 1 Secondary outcome measures assessing comorbidities and eating disorder associated behaviors.

## Abbreviations

DSM-5: Diagnostic and Statistical Manual of Mental Disorders, Fifth Edition
ED: eating disorder
EDE-A: Eating Disorder Examination Questionnaire Adolescent version
EDE-Q: Eating Disorder Examination Questionnaire
IACP: Intensive Ambulatory Care Program

## Data Availability

Data sharing is not applicable to this study, as no data sets were generated or analyzed for the preparation of this manuscript.
