# Supplementary material for: An Intensive Ambulatory Care Program for Adolescents With Eating Disorders Combining In-Person and Web-Based Care: Protocol for a Single-Site Naturalistic Trial
Source: JMIR Res Protoc. 2022 Nov 2;11(11):e37420. doi: 10.2196/37420 (PMC9669884; doi:10.2196/37420)
Supplement: Multimedia Appendix 1 [file resprot_v11i11e37420_app1.docx]

## **Multimedia Appendix**

# An Intensive Ambulatory Care Program for Adolescents with Eating Disorders Combining In-Person and Virtual Care: Protocol for a Naturalistic Feasibility, Acceptability, and Preliminary Effectiveness Trial

Kaylee Novack, Rachel Dufour, Louis Picard, Linda Booij, Nicholas Chadi

This is a Multimedia Appendix to a full manuscript published in the J Med Internet Res. For full copyright and citation information see <http://dx.doi.org/10.2196/jmir.37420>.

Secondary outcome measures assessing comorbidities and eating disorder associated behaviors:

The ***Affective Reactivity Index*** (ARI) [50] is a 6-item self-report questionnaire that evaluates chronic irritability based on 3 component measures: threshold for an angry reaction, frequency of angry feelings/behaviors, and the duration of such feelings/behaviors over the past 6 months. This measure was chosen as it is simple, concise and can be completed by self-report, the option retained for our project, though it has also been used with parents as well [50]. The questionnaire has good internal consistency (Cronbach α between 0.88 and 0.90 for self-reports) [50]. A French translation is available from the authors of the original survey. It was validated in a number of adolescent samples [49, 65]and in children and adolescents with clinical levels of anxiety [66].

The ***Child and adolescent perfectionism scale*** (CAPS) [67] is a 22-item self-report questionnaire that evaluates trait perfectionism. Two aspects of this trait are measured in the questionnaire: self-oriented perfectionism, which is a measure of an individual’s motivation and effort to be a perfectionist, and socially prescribed perfectionism, which is a measure of an individual’s belief about demands put upon them by others to be perfect [68]. This scale was chosen because it is one of the most used questionnaires evaluating this trait [34, 68]. The questionnaire has good internal consistency overall (Cronbach α 0.87) and for both subscales—i.e., the self-oriented (Cronbach α 0.83) and socially prescribed (Cronbach α 0.84) perfectionism measures [51, 67]. The English version is validated in several adolescent samples [67, 69] and in a sample of adolescents with anorexia nervosa [71]. The questionnaire was translated to French and validated in a sample of 145 adolescents (aged 10-17 years).

The ***Patient Health Questionnaire for Adolescents*** (PHQ-A) [72] is a modified version of the PHQ [73], a 9-item screening questionnaire for depression, intended specifically for adolescents that can be completed by self-report or administered by a clinician. It can be used for evaluating the severity of depressive symptoms. The adolescent version demonstrated satisfactory sensitivity, specificity and diagnostic agreement as compared to independent interviews with mental health professionals [72]. The questionnaire had good internal consistency in a sample of 846 adolescents in grades eight through twelve (Cronbach α 0.86) [52]. Several translations of the PHQ and PHQ- 9 exist, including the version available on the Pfizer Patient Health Questionnaire Screeners website [74], though no information about the process is available and we are not aware of studies that have validated French versions of the PHQ-9 in adolescent samples. Nonetheless, scores on the English and French versions of the PHQ-9 have been shown to be equivalent [75]. For this study, we will be using the French version of the PHQ-9, translated by *l’Institut Nationale d’Excellence en Santé et en Services Sociaux* (INESS) [75].

The ***Revised Children’s anxiety and depression scale*** (RCAD) [76] is a 47-item self-report questionnaire that was chosen because it evaluates multiple dimensions of anxiety: separation anxiety, social phobia, generalized anxiety disorder, panic disorder, obsessive compulsive disorder, and major depressive disorder. It is intended for use by children and adolescents between the ages of 9-18 years. Internal consistency of the questionnaire is high (Cronbach α 0.75 to 0.95) [53]. The English version has been validated in adolescents [77-78]. Clinical cut-offs have been reported, based on a study in a clinical sample [79]. A French translation of the questionnaire has been made available by the original developers on the Child First website [80]. This French version was validated in a sample of 704 adolescents [54].

The ***Generalized Anxiety Disorder – 7*** (GAD-7) [57] is a 7-item self-report questionnaire that measures the presence of generalized anxiety. Cut-off measurements have been established for presence or absence of GAD, in addition to symptom severity levels (mild-moderate-severe). The questionnaire was validated among adolescents and has acceptable specificity and sensitivity for detecting clinically significant anxiety symptoms in this population [81]. The questionnaire has excellent internal consistency (Cronbach α 0.92) and good test-retest reliability [57]. A French translation is available from the Pfizer Patient Health Questionnaire Screeners website [74] and has been validated in a sample of adolescents [55] and in people with epilepsy [56].

The ***Adolescent coping Scale*** (ACS) [58] is a 79-item self-report questionnaire intended specifically for adolescents 12-18 years old that evaluates 18 coping strategies, which can be grouped into three distinct subscales, each describing a particular coping style: Productive, Non-productive, and Reference to others. The internal consistency of the subscales of this measure were acceptable (Cronbach α 0.62-0.75) [58]. The questionnaire was translated to French, using a back-translation method. Internal consistency of the subscales of the translated measures were good (Cronbach α 0.83, 0.91, and 0.85 for the productive, non-productive, and reference to other subscales, respectively, for the non-clinical group and 0.85, 0.90, and 0.85 for the productive, non-productive, and reference to other subscales, respectively, in the clinical group [59]). This measure will only be used during the patient’s intake assessment.

The ***Self Esteem Rating Scale, short form*** (SERS-SF) [82] is a 20-item self-report questionnaire that evaluates two dimensions of self-esteem: positive and negative. It was chosen for its ability to measure changes in self-esteem over time. The original version has excellent internal consistency (Cronbach α 0.975) [82]. Lecomte et al. [60] translated the questionnaire to French using back-translation. This version had good internal consistency in a non-clinical sample of university students (Cronbach α 0.91 for the positive scale and 0.87 for the negative scale) [60]. Lecomte et al. [60] created a short form (SERS-SF). Scores on the short version were highly correlated with the Rosenberg Self-Esteem Scale, suggesting adequate convergent validity. Finally, confirmatory factor analysis confirmed good construct validity for the questionnaire, in both clinical and non-clinical samples [60].

The ***Eating Disorder Recovery Self-efficacy Questionnaire-f*** (EDRSQ-f) [83] is a 23-item self-report questionnaire that measures respondents’ confidence with regards to their recovery process using two scales: (1) Normative Eating Self-Efficacy (NESE), which is the confidence to eat without engaging in eating disordered behavior and (2) Body Image Self-Efficacy (BISE), which is the confidence to maintain a realistic body image that is not dominated by pursuit of thinness [61]. A French version has been validated in a sample of 203 university students, with a mean age of 21.8 years [62]. The translated questionnaire had excellent internal consistency (Cronbach α of 0.96 and 0.91 for the NESE and BISE scales, respectively), similar to the original version (Cronbach α of 0.97 and 0.95 for the NESE and BISE scales, respectively) [83]. Test-retest reliability for both scales were good (Pearson correlations 0.91 & 0.89) [62].

The ***Dépistage/Évaluation du Besoin d’Aide - Internet*** (DEBA-Internet) [84] is a 15-item self-report questionnaire that detects problematic internet usage and describes frequency of use of various internet platforms. The questionnaire explores various facets of use including usage habits, time devoted to usage, pursuit of usage despite fatigue, usage for the purpose of managing negative emotions, loss of control of usage, and perception of usage by one's friends and significant other. This tool was chosen for ease of interpretation (as the test is binary, any score passing a predetermined threshold indicates problematic usage) and because it is validated for use in those 12 years of age and older. The questionnaire has adequate sensitivity (0.82) and specificity (0.88) for detecting problematic internet and screen usage [63]. An English and French version are available from the developers. For this study, we chose to use only the first six questions of the DEBA-I and three additional questions were added to collect specific information about internet use in the context of eating disorders—i.e., using the internet to get information about nutritional values, exercise or caloric expenditures as well as diets and weight loss. This measure will be administered at patient intake.

The **Hyperactivity questionnaire** is a 3-item questionnaire created by members of our research team based on recommendations from Mantilla et al. [85] regarding questions that are helpful in evaluating and collecting information about eating disorder risks among adolescents who engage in compulsive exercise. Mantilla’s team identified items from the EDE-Q [64] and the Structural Analysis of Social Behavior (SASB), a self-report questionnaire relating to self-image with good psychometric properties [86-87], which were correlated with compulsive exercise behaviors in a sample of 482 adolescents (male and female) aged 12-15 years old. Based on this data, they suggested questions which can help clarify the intentions behind excessive/compulsive exercising in adolescents at risk of eating disorders. Two members of our research team, a clinical psychologist (LP) and a psycho-educator (VS) adapted these recommendations and created a self-report questionnaire and translated these questions to French. Psychometric properties are unknown.

The **Family Connectedness questionnaire** is a 6-item questionnaire created by our research team based on Olson’s Circumplex model [88], which proposes an evaluation of familial functioning based on three dimensions: (1) cohesion, that is, the emotional ties between family members, (2) flexibility, that is, the quality and expression of leadership and organisation of roles, rules, and negotiations, which varies from disengagement to relational entanglement where the optimal position is balanced cohesion and (3) communication, that is, the facilitating dimension in the face of family challenges and regulation of stressful experiences [88]. The questionnaire also uses recommendations from Pauzé & Pépitas [89], which proposes methods for evaluating family functioning based on an extensive review of the literature on the topic. The dimensions measured in our questionnaire are the same as those measured in many standardized family functioning questionnaires, including the frequently used FACES-IV [90]. The advantages of this adaptation, however, over other standardized questionnaires include the evaluation of the same dimensions in a shorter format and the possibility for both the adolescent and the parent to complete the questionnaire, allowing for the evaluation of congruence between these two reports (though for our study, only adolescent data will be collected). Psychometric properties are unknown.

**Acceptability** of the intervention will be evaluated on a weekly basis (during all 4-6 weeks of the IACP) using short satisfaction questionnaires as well as at the end of the intervention using a final satisfaction survey distributed with the post-intervention measures. Questionnaires will be distributed to both participants and their parents. All satisfaction questionnaires were created specifically for this project and combine both 10-point Likert-scale (i.e., from “Not at all” to “Extremely”) and short-answer questions. The questionnaires were adapted by members of the multidisciplinary research team involved in this study including a graduate student in biomedical sciences (KN) and a clinical psychologist (LP) based on relevant literature [25, 91-92]. Satisfaction surveys will be distributed to adolescents and parents. In the short weekly questionnaires, separate questions will evaluate satisfaction with individual components and therapeutic activities (e.g., meal accompaniment sessions, individual and group sessions, etc.) experienced in the IACP. The post-intervention questionnaire will evaluate overall program satisfaction, using eight Likert-scale questions and one open-ended question, and satisfaction with the virtual mode of intervention, using seven Likert-scale questions and one open-ended question.

**Feasibility** of the intervention will be evaluated using several measures (see Table 3 for a full list of feasibility measures) collected by the IACP clinical staff. Recruitment rate will be estimated by calculating the number of eligible patients (who are enrolled to the IACP) and the number of patients who eventually enroll in the study. Retention rate will be calculated by dividing the number of participants who complete the post-intervention visit and measures by the number of participants who enrolled in the study. Adherence to protocol will be estimated both by 1) calculating the number of participants who complete at least one therapeutic session per week of treatment in the IACP and by 2) calculating the number of participants who complete at least one therapeutic session/week and complete all post-intervention measures. Reasons for dropping out of the intervention will be recorded by the clinical staff. Adherence to the intervention will be estimated using the number of hours of treatment attended (as recorded by the treating clinician) and the total number of hours of treatment offered at the onset of the intervention.
